# Pioglitazone Reduces β Amyloid Levels *via* Inhibition of PPARγ Phosphorylation in a Neuronal Model of Alzheimer’s Disease

**DOI:** 10.3389/fnagi.2019.00178

**Published:** 2019-07-17

**Authors:** Qiankun Quan, Yihua Qian, Xi Li, Ming Li

**Affiliations:** ^1^Department of Geriatrics, The Second Affiliated Hospital of Xi’an Jiaotong University, Xi’an, China; ^2^Department of Human Anatomy, Histology and Embryology, School of Basic Medical Sciences, Xi’an Jiaotong University Health Science Center, Xi’an, China; ^3^Key Laboratory of Environment and Genes Related to Diseases (Xi’an Jiaotong University), Ministry of Education of China, Xi’an Jiaotong University Health Science Center, Xi’an, China

**Keywords:** Alzheimer’s disease, pioglitazone, β amyloid, PPARγ phosphorylation, cyclin-dependent kinase 5, insulin-degrading enzyme, β-amyloid cleavage enzyme 1

## Abstract

It has been demonstrated that peroxisome proliferator-activated receptor γ (PPARγ) can regulate the transcription of its target gene, insulin-degrading enzyme (*IDE*), and thus enhance the expression of the IDE protein. The protein can degrade β amyloid (Aβ), a core pathological product of Alzheimer’s disease (AD). PPARγ can also regulate the transcription of other target gene, β-amyloid cleavage enzyme 1 (*BACE1*), and thus inhibit the expression of the BACE1 protein. BACE1 can hydrolyze amyloid precursor protein (APP), the precursor of Aβ. In adipose tissue, PPARγ agonists can inhibit the phosphorylation of PPARγ by inhibiting cyclin-dependent kinase 5 (CDK5), which in turn affects the expression of target genes regulated by PPARγ. PPARγ agonists may also exert inhibitory effects on the phosphorylation of PPARγ in the brain, thereby affecting the expression of the aforementioned PPARγ target genes and reducing Aβ levels. The present study confirmed this hypothesis by showing that PPARγ agonist pioglitazone attenuated the neuronal apoptosis of primary rat hippocampal neurons induced by Aβ_1–42_, downregulated CDK5 expression, weakened the binding of CDK5 to PPARγ, reduced PPARγ phosphorylation, increased the expression of PPARγ and IDE, decreased the expression of BACE1, reduced APP production, and downregulated intraneuronal Aβ_1–42_ levels. These effects were inhibited by the PPARγ antagonist GW9662. After CDK5 silencing with CDK5 shRNA, the above effect of pioglitazone was not observed, except when upregulating the expression of PPARγ in Aβ_1–42_ treated neurons. In conclusion, this study demonstrated that pioglitazone could inhibit the phosphorylation of PPARγ *in vitro* by inhibiting CDK5 expression, which in turn affected the expression of PPARγ target genes *Ide* and *Bace1*, thereby promoting Aβ degradation and reducing Aβ production. This reduced Aβ levels in the brain, thereby exerting neuroprotective effects in an AD model.

## Introduction

Excessive accumulation of β amyloid (Aβ) in the brain is a key pathological change in Alzheimer’s disease (AD; Tanzi et al., 2004), which can lead to neurofibrillary tangles (Bloom, 2014), inflammation (McGeer and McGeer, 2013), synaptic dysfunction (Skaper et al., 2017), oxidative stress (Butterfield et al., 2013), and neuronal apoptosis (Saad et al., 2015). Therefore, clearance of Aβ may help ameliorate the disease state of AD (Tanzi et al., 2004). Recent clinical and experimental studies have made considerable progress in investigating peroxisome proliferator-activated receptor γ (PPARγ) agonists for the treatment of AD. For example, treatment with pioglitazone reduced the levels of extracellular Aβ_1–42_ in culture media of Chinese hamster ovary cells transfected with mouse amyloid precursor protein (APP) 695 (Chang et al., 2015). Pioglitazone reduced the elevated levels of Aβ in the brain in a rat model of insulin resistance (Luo et al., 2011) and improved learning and memory function in transgenic animals with AD (Papadopoulos et al., 2013). Rosiglitazone reduced the levels of Aβ_1–40_ and Aβ_1–42_ in the cerebral cortex of transgenic mice with AD, reduced Aβ plaques and tau phosphorylation, rescued memory impairment (Escribano et al., 2010), and prevented Aβ-induced inflammation (Xu et al., 2014), mitochondrial dysfunction, and oxidative stress (Chiang et al., 2016). The cognitive function of patients with AD improved significantly after treatment with orally administered rosiglitazone for 24 weeks (Watson and Craft, 2003; Risner et al., 2006). Nevertheless, the mechanisms of action of PPARγ agonists in reducing Aβ remain unclear.

PPARγ is a ligand-activated nuclear transcription factor. When activated by its ligand, PPARγ binds to the peroxisome proliferator responsive element (PPRE) in the promoter region of its target genes, thereby regulating their expression (Houseknecht et al., 2002). It has been demonstrated that PPARγ can regulate the transcription of its target gene, insulin-degrading enzyme (*IDE*), by binding to the PPRE in the *IDE* promoter and enhancing the expression of the IDE protein (Du et al., 2009). PPARγ can also bind to the PPRE in the promoter of another target gene, β-amyloid cleavage enzyme 1 (*BACE1*), to inhibit the expression of the BACE1 protein (Lin et al., 2016). IDE degrades Aβ (Vingtdeux et al., 2015), whereas BACE1 is a key enzyme involved in the initiation of Aβ production that hydrolyzes the Aβ precursor, APP (Sadleir et al., 2015; Farajdokht et al., 2017). PPARγ agonists act on the cyclin-dependent kinase (CDK) 5 inhibitory pathway in adipose tissue, which inhibits the phosphorylation of the Ser273 site located in the hinge region between the DNA-binding domain and ligand-binding domain of the PPARγ in adipocytes, thereby affecting the expression of PPARγ target genes (Choi et al., 2010). Therefore, we hypothesized that PPARγ agonists may also exert inhibitory effects on the phosphorylation of PPARγ in the brain. We speculated that this effect was related to the inhibition of CDK5 and could affect the expression of the PPARγ target genes, *Ide* and *Bace1*, which in turn would reduce Aβ production and promote Aβ degradation, thereby exerting a neuroprotective effect.

## Materials and Methods

### Ethics Statement

All experimental procedures in this study were approved by the Ethics Committee of Xi’an Jiaotong University Health Science Center prior to initiation of the research work. Experiments were performed in accordance with the ethical guidelines of The Basel Declaration and the International Council for Laboratory Animal Science.

### Reagents

Pioglitazone, GW9662, and Aβ_1–42_ were purchased from Sigma, USA. Rabbit anti-rat polyclonal antibodies against IDE, BACE1, and APP were purchased from Abcam, UK. Rabbit anti-rat polyclonal antibodies against CDK5, PPARγ and Aβ_1–42_ were purchased from Wanleibio, Shenyang, China. Rabbit anti-rat polyclonal antibody against p-PPARγ-Ser273 was purchased from Bioss, Beijing, China. TUNEL assay kit, anti-β-actin antibody, and ECL reagent were purchased from Wanleibio, Shenyang, China. TRIzol, Super M-MLV reverse transcriptase, RNase inhibitor, and 2× Power Taq PCR MasterMix amplification reagent kit were purchased from BioTeke, Beijing, China. SYBR GREEN Master Mix was purchased from Solarbio, Beijing, China. Protein A sepharose beads were purchased from Santa Cruz, CA, USA.

### Isolation and Culture of Rat Hippocampal Neurons

Rat hippocampal neurons were cultured as described previously (Vadukul et al., 2017). Two-day-old Sprague–Dawley rats were decapitated. The hippocampal tissue was collected, cut into pieces, and trypsinized. DMEM containing 10% fetal bovine serum and penicillin-streptomycin was added to neutralize the digestive enzymes. After filtration and centrifugation, hippocampal neurons were plated into polylysine-coated six-well culture plates at a density of 5 × 10^5^/m, and cultured at 37°C in a saturated humidity atmosphere containing 5% CO_2_ for 8 h. After incubation, the medium was replaced with Neurobasal medium containing 2% B27, 0.5 mmol/L glutamine, 100 U/mL penicillin, and 100 U/mL streptomycin. After 48 h, 10 μmol/L of cytarabine was added to inhibit the growth of glial cells. Solutions were replaced with fresh ones every 3 days thereafter. Neurons were identified with anti-NeuN immunocytochemistry (Hamano et al., 2012), and purity of neurons was greater than 90%. The neurons were used for experiments after 14 days of culture (Vadukul et al., 2017).

### Drug Interventions

The primary neurons that had been cultured for 15 days were divided into four groups: control group, Aβ group, pioglitazone (Pio) group, and Pio + GW9662 group. The control group was not treated with any drug in the media. The Aβ group was treated with Aβ_1–42_ in the media to a final concentration of 8 μM (Yang et al., 2015) for 24 h. The Pio group was pretreated with pioglitazone (final concentration 10 μM; Wang et al., 2011) for 1 h, after which Aβ_1–42_ (final concentration 8 μM) was added for cotreatment for 24 h. The pioglitazone + GW9662 group was pretreated with GW9662 (final concentration 10 μM; Hamano et al., 2016) for 0.5 h and then with pioglitazone (final concentration 10 μM) for 1 h, after which Aβ_1–42_ (final concentration 8 μM) was added for cotreatment for 24 h.

### CDK5 RNA Interference

Oligonucleotide primers for CDK5 short hairpin RNA (shRNA) and scramble shRNA were synthesized by Wanleibio Co., Limited (Shenyang, China) according to the target sequence for CDK5 RNA interference which was designed based on the sequence of rat C*dk5* (GenBank: NM_080885.1) and the control sequence without interference effect respectively. The primer sequences were as follows: CDK5 shRNA forward primer 5′ccggcccGGAGAGACCTGTT7GCAGAAttcaagagaTTCTGCAACAGGTCTCTCCttttt3′, reverse primer 5′aattaaaaaGGAGAGACCTGTTGC AGAAtctcttgaaTTCTGCAACAGGTCTCTCCggg3′; scramble shRNA forward primer 5′ccggcccTTCTCCGAACGTGTCAC GTttcaagagaACGTGACACGTTCGGAGAAttttt3′, reverse primer 5′aattaaaaaTTCTCCGAACGTGTCACGTtctcttgaaACGTGACACGTTCGGAGAAggg3′. The corresponding double-stranded DNA was obtained by annealing the primers. Then the DNA was cloned into lentivirus vector. Then lentivirus packaging was performed to construct the lentiviral vector expressing CDK5 shRNA and the lentiviral vector expressing scramble shRNA. In the present article, the lentiviral vectors will be referred to as LV-CDK5-shRNA and LV-scramble-shRNA, respectively. The cultured primary neurons were divided into four groups: Aβ group, scramble group, CDK5 shRNA group and CDK5 shRNA+Pio group. Neurons in the Aβ group were treated with Aβ_1–42_ (final concentration 8 μM) for 24 h. The neurons in Aβ group were treated with Aβ_1–42_ (final concentration 8 μM) for 24 h. The neurons of the scramble and CDK5 shRNA group were infected with LV-scramble-shRNA and LV-CDK5-shRNA, respectively for 24 h, after which Aβ_1–42_ (final concentration 8 μM) was added for cotreatment for 24 h. The CDK5 shRNA+Pio group was infected with LV-CDK5-shRNA for 24 h and then with pioglitazone (final concentration 10 μM) for 1 h, after which Aβ_1–42_ (final concentration 8 μM) was added for cotreatment for 24 h.

### TUNEL Staining

Terminal deoxynucleotidyl transferase dUTP nick end labeling (TUNEL) staining was performed according to the manufacturer’s protocol. After the cells in each group had grown on the slides, 50 μL of 0.1% Triton X–100 were added and cells were left for 15 min at 25°C for permeabilization. After rinsing in phosphate-buffered saline (PBS), the TUNEL reaction solution was added. Cells were kept moisturized and incubated in the dark for 60 min at 37°C. The TUNEL reaction solution was prepared using the Enzyme solution and Label Solution at a 1:9 ratio. After rinsing in PBS, DAPI was used for counterstaining for 5 min in the dark. Cells were then rinsed with PBS. Subsequently, a fluorescent quencher was added, and the plates were sealed. Neurons were counted using fluorescence microscopy at a ×400 magnification (BX53, OLYMPUS, Japan) by two pathologists blinded to the treatment condition, and the average count of four random fields of vision was used as the final result. The percentages of TUNEL-positive neurons were calculated. All neuronal nuclei were stained with DAPI and appeared as blue fluorescence. The nuclei of apoptotic neurons were stained with the TUNEL solution and appeared as green fluorescence.

### Flow Cytometry

The cells in each group were centrifuged and washed with PBS and then resuspended in 500 μL of Buffer binding solution. Next, 5 μL of Annexin V-FITC and 10 μL of Propidium Iodide were added. Cells were incubated for 15 min at 37°C in the dark. After incubation, cells were collected and washed. Apoptosis was measured using flow cytometry (Accuri C6, BD, USA). Apoptotic rate = [Number of cells with Annexin V (+) Propidium Iodide (−) [Number of cells with Annexin V (+) Propidium Iodide (+)]/Total Number of Cells × 100%.

### Co-immunoprecipitation

Co-immunoprecipitation was carried out according to a previously described method (Rai et al., 2014). Cell lysate was obtained by incubating primary rat hippocampal neurons on ice with cold RIPA buffer, followed by addition of protein A sepharose bead for 30 min at 4°C. Then, the cell lysate was centrifuged at 14,000 rpm for 15 min. Next, the supernatant was incubated with anti-PPARγ antibody (1:500) and IgG antibody or anti-CDK5 antibody (1:500) and IgG antibody for 12 h at 4°C. Samples were then incubated with protein A sepharose beads at 4°C for 4 h. Protein A sepharose bead–antigen–antibody complexes were precipitated by centrifugation at 14,000 rpm and 4°C for 5 s and washed four times with cold PBS, followed by protein separation on SDS–PAGE. Separated proteins were used for Western blot for CDK5 or PPARγ.

### Western Blot

The neurons in each group were washed with ice-cold PBS three times to eliminate the excess exogenously added Aβ_1–42_. Total protein was extracted according to the manufacturer’s protocol. Protein concentrations were determined using the BCA protein assay. Proteins were heated at 95°C for denaturation and then separated by electrophoresis using an 8% SDS-PAGE gel. After separation, proteins were transferred to a nitrocellulose filter membrane. The membrane was then washed with TBST and blocked with 5% skim milk powder (diluted in TBST) for 1 h at 37°C. Then, the respective rabbit anti-rat polyclonal antibodies against CDK5 (1:500), p-PPARγ-Ser273 (1:400), PPARγ (1:500), IDE (1:1,000), BACE1 (1:500), APP (1:1,000), and Aβ_1–42_ (1:400) were added. After overnight incubation at 4°C, the membrane was washed with TBST. HRP-labeled secondary antibody (goat anti-rabbit, 1:5,000) was added and incubated for 45 min at 37°C, followed by TBST washes. ECL reagent was added for film development. The film was scanned and the optical density values of the target bands were analyzed using the Gel-Pro-Analyzer software (version 4.0, Media Cybernetics, USA).

### Real-Time PCR

Total RNA was extracted from the neurons in each group using Trizol, and RNA concentration was determined using ultraviolet spectrophotometry. Reverse transcription reaction was performed according to the manufacturer’s protocol. RNA, oligo (dT)_15_, random primers, and ddH_2_O were added to nuclease-free centrifuge tubes on an ice bath. Then tubes were heated to 70°C for 5 min, followed by rapid cooling on ice for 2 min. The reaction solution was collected after centrifugation and 2 μL of dNTP (2.5 mM each), 4 μL of 5× Buffer, and 0.5 μL of RNase inhibitor were added. Then, 1 μL (200 U) of Super M-MLV reverse transcriptase was added and the solution was gently mixed. The solution was incubated at 25° for 10 min and then at 42°C for 50 min. The solution was then heated to 80°C for 10 min to halt the reaction. The resulting cDNA was stored at −20°C. The mRNA sequences for rat CDK5, PPARγ, IDE, BACE1, and APP were obtained from the Genbank database. The corresponding primers were designed with Primer Premier 5.0 and synthesized by Sangon Biotech (Shanghai) Co., Limited in China. The primer sequences were as follows: CDK5 forward primer 5′GGACACCGACTGAGGAAC3′, reverse primer 5′TTGGGCACGACATTCAC3′, 103 bp; PPARγ forward primer 5′TACCACGGTTGATTTCTC3′, reverse primer 5′AATAATAAGGCGGGGACG3′, 155 bp; IDE forward primer 5′TCCCGTGAAGCGACTGT3′, reverse primer 5′GACTTGTCCGTGGTGGG3′, 180 bp; BACE1 forward primer 5′TCCGCATCACCATCCTT3′, reverse primer 5′TGACCGCTCCCATAACG3′, 123 bp; APP forward primer 5′ACTCTGTGCCAGCCAATA3′, reverse primer 5′TGAATCATGTCCGAACTCC3′, 158 bp; β-actin forward primer 5′GGAGATTACTGCCCTGGCTCCTAGC3′, reverse primer 5′GGCCGGACTCATCGTACTCCTGCTT3′, 155 bp. Real-time fluorescence-based quantitative PCR reactions were performed according to the manufacturer’s protocol of the 2× Power Taq PCR MasterMix amplification reagent kit. The reaction system comprised 1 μL of cDNA, 0.5 μL of upstream primer (10 μM), 0.5 μL of downstream primer (10 μM), and 10 μL of SYBR GREEN Master Mix brought to 20 μL with ddH_2_O. The reaction conditions were predenaturation at 94°C for 5 min followed by 40 cycles of the following: denaturation at 94°C for 10 s, annealing at 60°C for 20 s, and extension at 72°C for 30 s. The above reactions were performed in an Exicycler™ 96 Real-Time PCR machine (Exicycler 96, BIONEER, Korea). Relative mRNA expression levels were calculated using the 2^−ΔΔCt^ value formula (Mandrekar-Colucci et al., 2012).

### Data Analyses

All data are presented as mean ± SEM. Statistical analyses were performed using SPSS 13.0 statistical software. One-way analysis of variance was used for intergroup comparisons. *Post hoc* LSD *t-test* was used for comparison between two groups after analysis of variance. *P* < 0.05 was considered statistically significant.

## Results

### Effects of Pioglitazone on Aβ_1–42_ Induced Apoptosis in Rat Hippocampal Neurons

**Figure 1:**
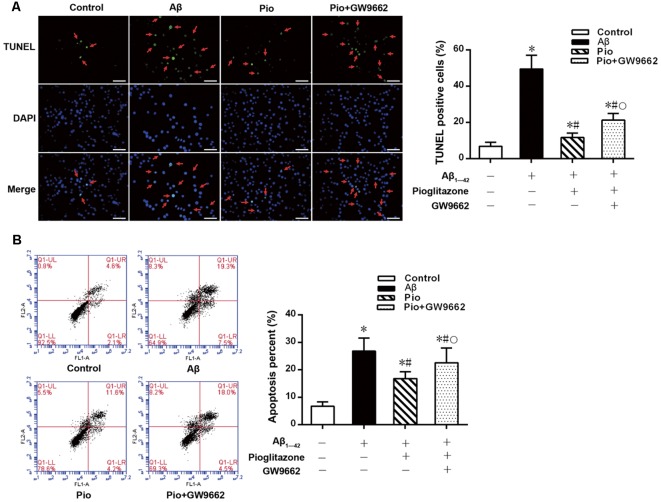
Effects of pioglitazone on β amyloid (Aβ)_1–42_ induced apoptosis in rat hippocampal neurons. **(A)** Results of Terminal deoxynucleotidyl transferase dUTP nick end labeling (TUNEL) staining. Green represents nuclei with positive TUNEL staining (indicated by red arrows), and blue represents nuclei counterstained with DAPI. Scale bar, 50 μm. **(B)** Results of flow cytometry. *n* = 6 wells per group. *P < 0.05 compared to the control group, ^#^*P* < 0.05 compared to the Aβ group, °*P* < 0.05 compared to the Pio group.

**Figure 2:**
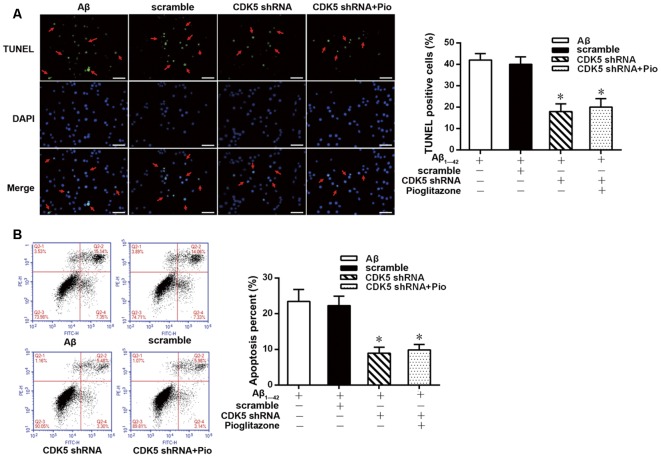
Effects of pioglitazone on Aβ_1–42_ induced apoptosis in rat hippocampal neurons after cyclin-dependent kinase 5 (CDK5) silencing. **(A)** Results of TUNEL staining. Green represents nuclei with positive TUNEL staining (indicated by red arrows), and blue represents nuclei counterstained with DAPI. Scale bar, 50 μm. **(B)** Results of flow cytometry. *n* = 6 wells per group. *P < 0.05 compared to the Aβ group.

TUNEL staining and flow cytometry were used to examine the effects of Aβ_1–42_ on the apoptosis of rat hippocampal neurons and the protective effects of pioglitazone. The results of TUNEL staining were consistent with that of flow cytometry. As the core pathological product of AD, Aβ_1–42_ significantly induced neuronal apoptosis, and the percentage of apoptotic neurons was higher than that of control group. The PPARγ agonist pioglitazone significantly reduced Aβ_1–42_ induced neuronal apoptosis. TUNEL-positive staining in neurons and the neuronal apoptotic rate from flow cytometry were significantly decreased, indicating that pioglitazone could counteract the toxicity caused by Aβ_1–42_ and exert a protective effect on neuronal injury. To investigate whether this effect of pioglitazone was related to PPARγ agonism, neurons were pretreated with the PPARγ antagonist GW9662 prior to intervention with Aβ_1–42_ and pioglitazone. GW9662 reversed the protective effect of pioglitazone, suggesting that the inhibitory effect of pioglitazone on neuronal apoptosis is related to PPARγ (Figure 1). It is established that PPARγ agonists can inhibit PPARγ phosphorylation induced by CDK5 in adipose tissue and that this effect can be inhibited by GW9662 (Choi et al., 2010). In order to verify whether pioglitazone acted directly on CDK5 or PPARγ to inhibit Aβ_1–42_ induced apoptosis of hippocampal neurons, we tested the effect of pioglitazone on neuronal apoptosis induced by Aβ_1–42_ after silencing CDK5 expression with CDK5 shRNA. Results showed that Aβ_1–42_ induced neuronal apoptosis was significantly attenuated after CDK5 silencing. However, pioglitazone treatment after CDK5 silencing did not further improve the attenuation of neuronal apoptosis induced by CDK5 silencing (Figure 2). This suggests that pioglitazone may inhibit neuronal apoptosis by directly acting on CDK5 rather than PPARγ.

### Effects of Pioglitazone on Intraneuronal Aβ_1–42_ Levels in AD Model Neurons

**Figure 3:**
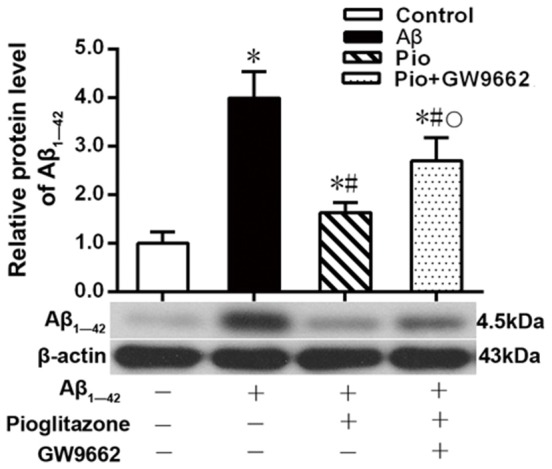
Effects of pioglitazone on the levels of Aβ_1–42_ in Alzheimer’s disease (AD) model neurons. *n* = 6 wells per group. *P < 0.05 compared to the control group, ^#^*P* < 0.05 compared to the Aβ group, °*P* < 0.05 compared to the Pio group.

**Figure 4:**
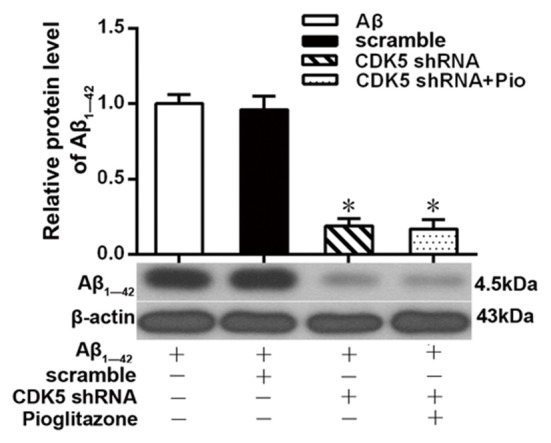
Effects of pioglitazone on the levels of Aβ_1–42_ in AD model neurons after CDK5 silencing. *n* = 6 wells per group. *P < 0.05 compared to the Aβ group.

Aβ plays a key role in the pathogenesis of AD and can lead to neuronal apoptosis (Farajdokht et al., 2017). Since Aβ_1–42_ is one of the main components of Aβ (Selkoe, 2001), we tested the effects of pioglitazone on Aβ_1–42_ levels in hippocampal neuronal cultures. As shown in Figure 3, the addition of Aβ_1–42_ to the culture medium significantly increased the levels of Aβ_1–42_ in rat hippocampal neurons, and treatment with pioglitazone effectively reduced the levels of Aβ_1–42_. Pretreatment with GW9662 prior to interventions with Aβ_1–42_ and pioglitazone attenuated the reduction of Aβ_1–42_ caused by pioglitazone, suggesting that PPARγ was involved in the reduction of Aβ_1–42_ induced by pioglitazone. Next, we silenced the expression of CDK5 and observed the effect of pioglitazone on the intraneuronal Aβ_1–42_ levels. The results showed that the intraneuronal Aβ_1–42_ levels increased significantly after CDK5 silencing, but pioglitazone treatment did not affect the increase of intraneuronal Aβ_1–42_ levels induced by CDK5 silencing (Figure 4). This suggests that pioglitazone may decrease Aβ_1–42_ levels by acting on CDK5 rather than PPARγ directly.

### Effects of Pioglitazone on PPARγ Target Gene Expression in AD Model Neurons

**Figure 5:**
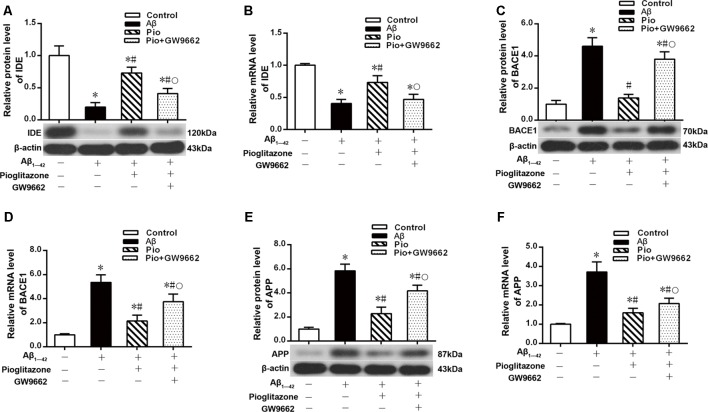
Effects of pioglitazone on peroxisome proliferator-activated receptor γ (PPARγ) target gene expression in AD model neurons. **(A)** Western blot results for insulin-degrading enzyme (IDE). **(B)** Real-time PCR results for IDE. **(C)** Western blot results for β-amyloid cleavage enzyme 1 (BACE1). **(D)** Real-time PCR results for BACE1. **(E)** Western blot results for amyloid precursor protein (APP). **(F)** Real-time PCR results for APP. *n* = 6 wells per group. *P < 0.05 compared to the control group, ^#^*P* < 0.05 compared to the Aβ group, °*P* < 0.05 compared to the Pio group.

**Figure 6:**
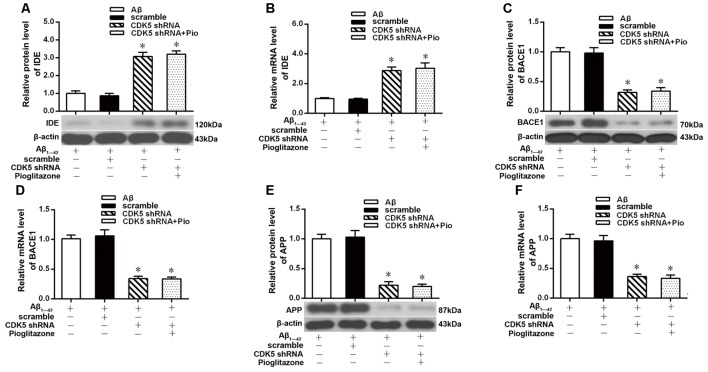
Effects of pioglitazone on PPARγ target gene expression in AD model neurons after CDK5 silencing. **(A)** Western blot results for IDE. **(B)** Real-time PCR results for IDE. **(C)** Western blot results for BACE1. **(D)** Real-time PCR results for BACE1. **(E)** Western blot results for APP. **(F)** Real-time PCR results for APP. *n* = 6 wells per group. *P < 0.05 compared to the Aβ group.

We next investigated the mechanism by which pioglitazone reduced neuronal Aβ_1–42_ levels. The PPARγ target gene *Ide* is involved in the degradation of Aβ (Vingtdeux et al., 2015), and another target gene, *Bace1*, is involved in the hydrolysis of the Aβ precursor, APP (Sadleir et al., 2015; Farajdokht et al., 2017). Therefore, we evaluated the effects of the PPARγ agonist pioglitazone and the PPARγ antagonist GW9662 on these genes. Treatment with Aβ_1–42_ in neuronal cultures decreased the protein and mRNA levels of IDE compared to those of control neurons, whereas the protein and mRNA levels of BACE1 and APP were increased compared with those of control neurons. Treatment with pioglitazone in neurons treated with Aβ_1–42_ increased the expression of IDE protein and mRNA and decreased the expression of BACE1 and APP proteins and mRNA. Pretreatment with GW9662 in neurons treated with Aβ_1–42_ and pioglitazone decreased the expression of IDE and increased the expression of BACE1 and APP. These results suggested that pioglitazone upregulated IDE expression and downregulated BACE1 and APP expression through PPARγ, which promoted Aβ degradation and reduced Aβ production (Figure 5). Silencing CDK5 expression with CDK5 shRNA could significantly inhibit the downregulation of IDE and upregulation of BACE1 and APP induced by Aβ_1–42_. Pioglitazone treatment after CDK5 silencing did not strengthen or weaken the above effects induced by CDK5 silencing (Figure 6).

### Effects of Pioglitazone on CDK5 Expression and PPARγ Phosphorylation in AD Model Neurons

**Figure 7:**
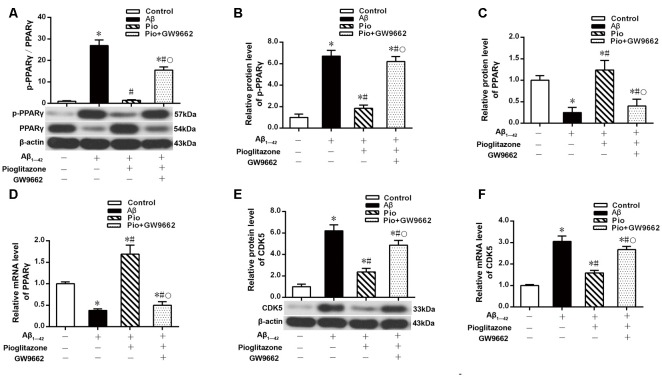
Effects of pioglitazone and Aβ_1–42_ on PPARγ phosphorylation in AD model neurons. **(A)** Intergroup comparisons of p-PPARγ/PPARγ ratios. **(B)** Intergroup comparisons of the p-PPARγ protein levels. **(C)** Intergroup comparisons of PPARγ protein levels. **(D)** Intergroup comparisons of PPARγ mRNA levels. **(E)** Intergroup comparisons of CDK5 protein levels. **(F)** Intergroup comparisons of CDK5 mRNA levels. *n* = 6 wells per group. *P < 0.05 compared to the control group, ^#^*P* < 0.05 compared to the Aβ group, °*P* < 0.05 compared to the Pio group.

**Figure 8:**
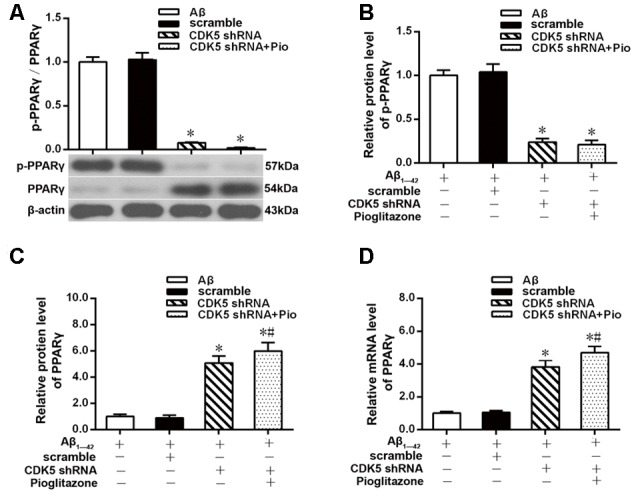
Effects of pioglitazone and Aβ_1–42_ on PPARγ phosphorylation in AD model neurons after CDK5 silencing. **(A)** Intergroup comparisons of p-PPARγ/PPARγ ratios. **(B)** Intergroup comparisons of the p-PPARγ protein levels. **(C)** Intergroup comparisons of PPARγ protein levels. **(D)** Intergroup comparisons of PPARγ mRNA levels. *n* = 6 wells per group. *P < 0.05 compared to the Aβ group, ^#^*P* < 0.05 compared to the CDK5 shRNA group.

**Figure 9:**
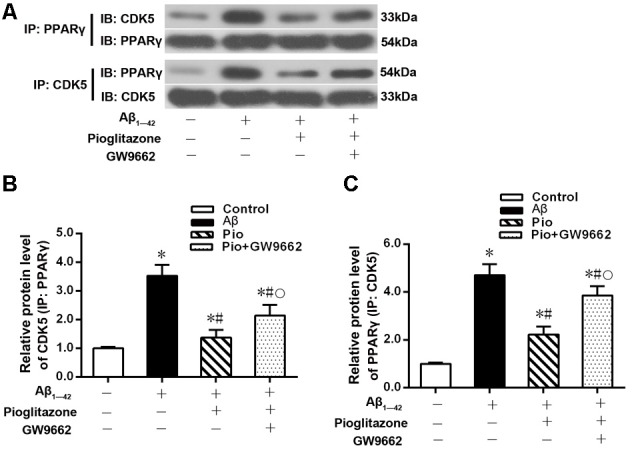
Effects of pioglitazone and Aβ_1–42_ on the binding of CDK5 to PPARγ in AD model neurons. **(A)** Representative immunoblots to show reciprocal co-IP experiments in using anti-CDK5 or anti-PPARγ antibodies. **(B)** Intergroup comparisons of CDK5 protein levels in immunoprecipitated complexes obtained using anti-PPARγ antibody. **(C)** Intergroup comparisons of PPARγ protein levels in immunoprecipitated complexes obtained using anti-CDK5 antibody. *n* = 6 wells per group. *P < 0.05 compared to the control group, ^#^*P* < 0.05 compared to the Aβ group, °*P* < 0.05 compared to the Pio group.

The above experiments indicated that CDK5 and PPARγ are involved in the protective effect of pioglitazone on neurons treated with Aβ_1–42_ and in the reduction of Aβ_1–42_ levels. Next, we investigated the effects of Aβ_1–42_ and pioglitazone on CDK5 expression and PPARγ phosphorylation in primary hippocampal neurons. Results showed that Aβ_1–42_ decreased the protein levels and mRNA expression of PPARγ and increased the phosphorylation of PPARγ at Ser273 site, which increased the p-PPARγ/PPARγ ratio. Pioglitazone significantly inhibited the PPARγ phosphorylation induced by Aβ_1–42_ and upregulated the expression of PPARγ, which lowered the p-PPARγ/PPARγ ratio. The PPARγ antagonist GW9662 attenuated the effects of pioglitazone (Figures 7A–D). Additionally, we tested the effects of pioglitazone and Aβ_1–42_ on the expression of CDK5. As shown in Figures 7E,F, Western blot and real-time PCR revealed that Aβ_1–42_ upregulated the protein and mRNA expression of CDK5. Pioglitazone inhibited the effects of Aβ_1–42_ as it downregulated CDK5 protein and mRNA expressions. We also utilized the PPARγ antagonist GW9662 in this set of experiments. GW9662 inhibited the downregulation of CDK5 expression caused by pioglitazone. In order to further confirm whether pioglitazone affects the phosphorylation and expression of PPARγ through CDK5, we silenced CDK5 expression using CDK5 shRNA, and then tested the effect of pioglitazone on the phosphorylation status of PPARγ and its expression in primary rat hippocampal neurons induced by Aβ_1–42_. Results showed that CDK5 silencing significantly inhibited PPARγ phosphorylation and upregulated the expression of PPARγ in primary rat hippocampal neurons treated with Aβ_1–42_. Pioglitazone treatment after CDK5 silencing had no effect on the decrease in PPARγ phosphorylation induced by CDK5 silencing (Figures 8A,B), whereas it further strengthened the upregulation of PPARγ induced by CDK5 silencing (Figures 8C,D). These results suggest that pioglitazone may inhibit PPARγ phosphorylation by inhibiting CDK5 in AD model neurons. In order to further confirm this conclusion, we carried out a co-immunoprecipitation experiment. Results showed that pioglitazone effectively inhibited the enhanced binding of CDK5 to PPARγ induced by Aβ_1–42_ in primary rat hippocampal neurons and GW9662 effectively blocked the above effects of pioglitazone (Figure 9).

## Discussion

Using an *in vitro* AD model, this study demonstrated that PPARγ agonists pioglitazone inhibited the phosphorylation of PPARγ by inhibiting CDK5 expression. Here we provide a preliminary mechanism of action of PPARγ agonists for Aβ clearance. PPARγ is a ligand-activated nuclear transcription factor that positively regulates the expression of its target gene *Ide* (Lin et al., 2016) and negatively regulates the expression of *Bace1* (Lin et al., 2016). The former degrades Aβ (Li et al., 2015), whereas the latter hydrolyzes APP to produce Aβ (Sadleir et al., 2015). Phosphorylation is an important posttranslational modification of PPARγ. The phosphorylation sites in PPARγ differ depending on cytokines and stimuli, which results in different biological effects. Studies have shown that mitogen-activated protein kinases (MAPKs) in bone inhibit the activity of PPARγ by phosphorylation of PPARγ at Ser112 (Ge et al., 2016); CDK5 in adipose tissue inhibits the activity of PPARγ by phosphorylation of PPARγ at Ser273, leading to functional impairment and changes in expression of its target genes (Choi et al., 2010); CDK7 mediates the phosphorylation of PPARγ at Ser112, which inhibits the activity of PPARγ and suppresses adipogenesis (Helenius et al., 2009); and CDK9 modifies the phosphorylation of PPARγ at Ser112, which increases PPARγ activity and upregulates the expression of its target genes (Iankova et al., 2006). The aging process leads to an increase in PPARγ phosphorylation in various tissues, such as the cerebral cortex (Sanguino et al., 2006), kidney (Sung et al., 2004), bone (Ge et al., 2016), and adipose tissue (Ye et al., 2006). The results of the present study revealed that the expression of CDK5 was elevated in primary cultures of rat hippocampal neurons after treatment with Aβ_1–42_. The levels of PPARγ phosphorylation at Ser273 increased, and the expression of its downstream target genes was also affected. For instance, *Ide* expression was reduced whereas *Bace1* expression was elevated. In the adipose tissue of an obese mouse model induced by high-fat feeding, phosphorylation of PPARγ by CDK5 was inhibited by the PPARγ agonist rosiglitazone (Choi et al., 2010). Similarly, the present study indicated that PPARγ phosphorylation mediated by CDK5 was inhibited by a PPARγ agonist in an AD neuronal model. By using the PPARγ agonist pioglitazone, PPARγ antagonist GW9662 and CDK5 shRNA, the results of this study indicate that pioglitazone inhibits PPARγ phosphorylation by inhibiting the expression of CDK5, which in turn affects the expression of its target genes *Ide* and *Bace1*, reduces Aβ production, promotes Aβ degradation, and reduces neuronal apoptosis induced by Aβ, which exerted neuroprotective effects.

In the study, we found that pioglitazone increases the expression of PPARγ in neurons treated with Aβ_1–42_ after silencing CDK5 expression, which indicates that upregulation of PPARγ expression was not due to or not only due to the inhibition of CDK5 by pioglitazone, which probably due to the direct upregulation of expression of PPARγ induced by pioglitazone as PPARγ agonist. This needs to be further confirmed in the future. In theory, upregulation of PPARγ expression leads to change in the expression of PPARγ target genes. However, our study found that pioglitazone had no effect on the expression of IDE and BACE1 in neurons treated with Aβ_1–42_ after CDK5 silencing. We speculate that the reason for this is that CDK5 silencing has largely inhibited PPARγ phosphorylation and upregulated the expression of PPARγ, which affects the expression of PPARγ target genes to the maximum rate, so even if pioglitazone has a direct effect on the upregulation of PPARγ, the effect could not be accumulated. CDK5 is required to bind to P25, a truncated form of p35, to become activated (Patrick et al., 1999) and to phosphorylate PPARγ at the Ser237 site (Choi et al., 2010). The present study only investigated the effect of pioglitazone on CDK5 expression. Further studies are needed to elucidate whether pioglitazone also affects p35 and p25.

Interestingly, in this study, we found that the aforementioned effect of pioglitazone is partially reversed by GW9662, which suggests that there may be other mechanisms involved in the effect of pioglitazone in addition to acting on CDK5 and PPARγ. In addition to IDE, it is known that other intraneuronal proteases can also degrade intraneuronal Aβ, such as endothelin-converting enzyme (Pacheco-Quinto and Eckman, 2013), neprilysin (Turrel et al., 2017) and nuclear-inclusion-a (Shin et al., 2014). It is unclear whether these enzymes are involved in the reduction of Aβ induced by pioglitazone. Additionally, pioglitazone can also have a weak activation effect on PPARα (Sakamoto et al., 2000; Orasanu et al., 2008). An antagonist of this receptor, such as GW6471, could be used to check whether PPARα is also involved in the effect of pioglitazone on Aβ in future work. Furthermore, the partial reversal of pioglitazone by GW9662 may be due to an insufficient dose of GW9662 in the study. This seems to be a more reasonable explanation because 10 μM GW9662 did not completely reverse the up-regulation of PPARγ expression induced by pioglitazone. The issue could be verified by increasing the dose of GW9662 in the future.

We observed that the level of CDK5 increased in neurons treated with Aβ_1–42_. This result was inconsistent with previous findings indicating that CDK5 expression was unchanged in the brains of 6-month-old APP/PS1 transgenic mice and in rat hippocampal neurons treated with Aβ. On the contrary, an increase in CDK5 activity was observed (Chen et al., 2015). Since the AD models and Aβ doses utilized in these two studies were different, the experimental results are not directly comparable. Further investigations are required to address the discrepancies.

In addition, the present study showed that Aβ treatment resulted in decreased IDE expression and increased BACE1 and APP expression, consistent with other studies. Previous studies have shown that the level of IDE protein in the brain of AD patients is reduced, and the activity of IDE protein is negatively correlated with the level of Aβ_1–42_ (Pérez et al., 2000; Zhao et al., 2007). Injection of Aβ_1–42_ into the hippocampus reduced both mRNA and protein levels of IDE in the hippocampus in an AD rat model (Khodadadi et al., 2018). Aβ_1–42_ treatment increased the level of BACE1 and APP in primary mouse neuronal cultures (Sadleir et al., 2014). In addition, increased levels of BACE1 and APP also have been observed in the cortex and hippocampus of 5XFAD transgenic mice (Sadleir et al., 2014). In the present study, we noticed a simultaneous increase in CDK5 expression and PPARγ phosphorylation. CDK5 has been previously shown to inhibit the activity of PPARγ by mediating the phosphorylation of PPARγ at Ser273 (Choi et al., 2010). Therefore, we hypothesized that Aβ may promote PPARγ phosphorylation by upregulating CDK5 expression, which in turn affected the expression of IDE and BACE1. In addition, the decrease in IDE expression may be caused by the exhaustion of IDE supply used for Aβ degradation.

Furthermore, a study suggests that exogenous Aβ can lead to elevated levels of BACE1 and APP, which may occur because Aβ causes primary neurons to become swollen and dystrophic, leading to the accumulation of BACE1 and APP (Sadleir et al., 2014). Further studies are required to elucidate the pathogenesis of AD and to determine whether changes in CDK5 expression or activity initiate Aβ production, whether pathological Aβ affects CDK5, or whether both phenomena interact.

In this study, the addition of Aβ_1–42_ to the culture medium resulted in a significant increase in intracellular levels of Aβ in neurons, which was consistent with the results of some studies (Yang et al., 2015; Gwon et al., 2018). It has been proved that Aβ can be taken up from extracellular sources through receptor binding and subsequent internalization (LaFerla et al., 2007). This seems to be a reasonable explanation for the elevated level of intraneuronal Aβ caused by treatment with exogenous Aβ. Receptors to which Aβ can bind include α7 nicotinic acetylcholine receptor (Nagele et al., 2002), low-density lipoprotein receptor-related protein (Harris-White et al., 2004), the receptor for advanced glycation end products (Lana et al., 2017), p75 neurotrophin receptor (Ovsepian and Herms, 2013), Fcγ-receptor IIb (Gwon et al., 2018).

Both extracellular Aβ and intracellular Aβ play an essential role in the onset of AD (LaFerla et al., 2007). It is generally thought that extracellular Aβ is generated by BACE1-mediated APP proteolysis and released in the extracellular matrix, which is a major cause of AD development (Vassar et al., 1999; Zhao et al., 2019). APP proteolysis mediated by BACE1 can also occur in the endoplasmic reticulum (Jang et al., 2017), Golgi apparatus (Toh et al., 2017) and endosomes (Furusawa et al., 2019) to produce intracellular Aβ. Furthermore, Aβ can be taken up from extracellular sources through subsequent internalization (LaFerla et al., 2007; Ripoli et al., 2014). Intracellular Aβ accumulation appears to be an early event in AD because it precedes the deposition of both neurofibrillary tangles and senile plaques (Gouras et al., 2000; LaFerla et al., 2007; Welikovitch et al., 2018). Intracellular Aβ accumulation can markedly affect glutamatergic synaptic transmission and plasticity (Ripoli et al., 2014), cognitive impairment (Iulita et al., 2014), neuronal death (Umeda et al., 2011), inflammatory signs and tau phosphorylation (Rebeck et al., 2010), and mitochondrial dysfunction (Zepa et al., 2011). It has been shown that intracellular Aβ can be effectively degraded by cytosolic IDE (Stargardt et al., 2013). In the present study, we observed that pioglitazone can reduce intraneuronal Aβ levels *via* inhibition of PPARγ phosphorylation, which functions *via* inhibition of CDK5 expression, thereby upregulating the expression of IDE and downregulating the expression of BACE1 in a neuronal model of AD. We did not, however, investigate changes in extracellular Aβ levels. The reason for this is that following addition of exogenous Aβ, it is difficult to distinguish exogenous Aβ from endogenously produced Aβ that has been secreted into the culture medium.

## Conclusion

In conclusion, this study demonstrated that the PPARγ agonist pioglitazone inhibits the phosphorylation of PPARγ in a neuronal model of AD by inhibiting the CDK5 pathway. This reduced Aβ levels, thereby exerting a protective effect on neurons.

## Ethics Statement

This study was carried out in accordance with the recommendations of “the ethical guidelines of The Basel Declaration and the International Council for Laboratory Animal Science.” The protocol was approved by the “Ethics Committee of Xi’an Jiaotong University Health Science Center.”

## Author Contributions

QQ, YQ, and XL designed the study and contributed with important reagents. QQ, YQ, and ML performed the study. QQ and ML collected the data. QQ analyzed the data. QQ and YQ wrote the manuscript.

## Conflict of Interest Statement

The authors declare that the research was conducted in the absence of any commercial or financial relationships that could be construed as a potential conflict of interest.
